# Single and Mixture Toxicity of Boron and Vanadium Nanoparticles in the Soil Annelid *Enchytraeus crypticus*: A Multi-Biomarker Approach

**DOI:** 10.3390/nano12091478

**Published:** 2022-04-27

**Authors:** Ana Capitão, Joana Santos, Angela Barreto, Mónica J. B. Amorim, Vera L. Maria

**Affiliations:** Department of Biology & CESAM, University of Aveiro, 3810-193 Aveiro, Portugal; amcapitao@ua.pt (A.C.); joanasilvasantos@ua.pt (J.S.); abarreto@ua.pt (A.B.); mjamorim@ua.pt (M.J.B.A.)

**Keywords:** nanotoxicity, metal(loid) nanomaterials, combination of contaminants, terrestrial ecosystem, oxidative stress/damage, phenotypical endpoints, mixture toxicity

## Abstract

The increased use and production of new materials has contributed to Anthropocene biodiversity decrease. Therefore, a careful and effective toxicity evaluation of these new materials is crucial. However, environmental risk assessment is facing new challenges due to the specific characteristics of nanomaterials (NMs). Most of the available ecotoxicity studies target the aquatic ecosystems and single exposures of NMs. The present study evaluated *Enchytraeus crypticus* survival and reproduction (28 days) and biochemical responses (14 days) when exposed to nanoparticles of vanadium (VNPs) and boron (BNPs) (single and mixture; tested concentrations: 10 and 50 mg/kg). Although at the organism level the combined exposures (VNPs + BNPs) did not induce a different toxicity from the single exposures, the biochemical analysis revealed a more complex picture. VNPs presented a higher toxicity than BNPs. VNPs (50 mg/kg), independently of the presence of BNPs (additive or independent effects), caused a decrease in survival and reproduction. However, acetylcholinesterase, glutathione S-transferase, catalase, glutathione reductase activities, and lipid peroxidation levels revealed alterations in neurotoxicity, detoxification and antioxidant responses, depending on the time and type of exposure (single or mixture). The results from this study highlight different responses of the organisms to contaminants in single versus mixture exposures, mainly at the biochemical level.

## 1. Introduction

At the beginning of the 21st century nanoscience and nanotechnology started to gain exponential interest with consequent increase in the production and use of several nanomaterials (NMs) [1,2,3,4]. The increased use of engineered NMs gave rise to concerns regarding their environmental fates and behaviors [5,6]. While most toxicity research focuses on aquatic ecosystems, where the predicted concentrations of nanoparticles (NPs) are between ng/L and µg/L (depending on the NP), soil ecosystem contamination by NPs has been studied less [7]. However, NPs will reach and contaminate soil; for example, through wastewater biosolids application, waste discharging from industrialization processes and direct application in agriculture (for example: nano-fertilizers and nano-pesticides) [7,8,9,10]. Characteristics, such as high surface/volume ratio, that make NPs very attractive for industrial and medical applications, are the same that increment their toxicity and make their environmental risk assessment difficult [6,7,11]. Reaching the environment, NMs can be bioaccumulated and biomagnified through the food chain, achieving higher concentrations compared with ones found in the environment [12].

The diversity of NMs applications increases their potential ecosystem contamination, due to overuse or improper disposal [9]. Boron (B) and vanadium (V) NPs are examples of NPs with a wide range of applications, from industrial (fuels, electronics, batteries, etc.) to medical products (antimicrobial activity, drug delivery, etc.) [13,14,15]. Although the environmental concentration of B and V elements is known to be on average 10 mg/kg for B and 240 mg/kg for V [16,17], no information was found regarding the level of contamination with BNPs and VNPs. Studies focusing on the toxicity of these two types of NPs are scarce. However, those that are available reveal a higher toxicity of VNPs when compared with BNPs in soil and aquatic organisms [18,19]. VNPs were reported to cause oxidative stress with the increase of reactive oxygen species (ROS) and consequent DNA damage in lung cell lines in Wistar rats [20,21]. Additionally, in *Enchytraeus crypticus,* VNPs impaired survival and reproduction (concentration that causes 50% of the effect (EC_50_) = 11 mg/kg) [18] and in *Danio rerio* embryos induced malformations and a delay in hatching (10 mg/L) [19]. BNPs caused 100% mortality in *Daphnia magna* (concentration that causes 50% of mortality (LC_50_) = 6.7 mg/L) [22], a decrease in the reproduction of *E. crypticus* (EC_50_ = 319 mg/kg) [18] and induced malformations in *D. rerio* embryos (10 mg/L) [19]. Curiously, in bulk form, B (5–20 mg/L) prevented cellular damage, increasing the antioxidant response in *D. rerio* [23,24], and even reduced V oxidative damage in vitro (human lymphocytes) [25] and in watermelon roots [26].

Despite the increasing attention of the research community on the ecotoxicological scope of different NMs, several questions are still far from being answered, mostly for soil ecosystems. For example, reports of metal oxide NP nanotoxicity often describe the organisms’ production of ROS and subsequent damage of cell defense systems [4,9] and VNPs are no different. However, no data were found regarding BNPs. Will BNPs contribute to ROS production? Or will the presence of BNPs reduce the effects of VNPs as observed for the bulk form? Our work seeks to answer these questions and contribute to increasing the comprehension of the effects posed by single and combined NP (VNPs + BNPs) exposure. To this end, the soil annelid *E. crypticus* was selected. The enchytraeids are sensitive, versatile, ecologically relevant, present a high reproduction rate, a short life cycle and can be found in almost every terrestrial ecosystem [9,27]. With uptake routes that include the skin and the gut, *E. crypticus* have been widely used in soil nanotoxicology [18,28,29].

The interference of several NPs with antioxidant response mechanisms has been commonly reported. To verify if this mechanism is responsible for the reported decrease in the survival and reproduction of *E. crypticus* exposed to BNPs and VNPs [18], our study evaluates the alterations in key antioxidant enzymes after single and mixture exposure to BNPs and VNPs. Towards a more mechanistic approach, we also evaluated the effects of combination of both NPs.

## 2. Materials and Methods

### 2.1. Test Materials and Characterization

The test materials were acquired from Nanoshel UK Limited (Congleton, Cheshire, UK). BNPs (CAS: 7440-42-8; Stock No: NS6130-12-001263) and VNPs (CAS: 7440-62-2; No: NS6130-12-001065) were dispersed in 2% of Triton X-100 and water with an average particle size between 80–100 nm and a purity of 99.9%. The characteristics (hydrodynamic size (HS), zeta potential (ZP) and polydispersity index (PDI)) of the NPs’ test dispersions (performed in ultrapure water) were assessed using a Zetasizer Nano ZS, Malvern, UK.

### 2.2. Soil Invertebrates

The soil model annelid *Enchytraeus crypticus*, Westheide & Graefe, 1992 [27,30] was selected. Cultures were maintained in the laboratory in supplemented agar plates (20 ± 1 °C; 16 h:8 h light:dark photoperiod) and fed twice per week with ground-autoclaved oats as described by Barreto et al. [18].

### 2.3. Test Soil and Experimental Conditions

LUFA 2.2 standard natural soil (main characteristics in Table S1) was used for the tests and placed for 48 h at 60 °C to dry before assays. For Enchytraeid Reproduction Test (ERT), experimental conditions were selected based on the results of the study by Barreto and co-workers [18]. Test conditions included: control (C), solvent control (0.05% Triton X-100-SC), 10 mg BNPs/kg soil (10B), 50 mg BNPs/kg soil (50B), 10 mg VNPs/kg soil (10V), 50 mg VNPs/kg soil (50V), 10 mg VNPs + 10 mg BNPs/kg soil (10V + 10B), 10 mg VNPs + 50 mg BNPs/kg soil (10V + 50B), 50 mg VNPs + 10 mg BNPs/kg soil (50V + 10B) and 50 mg VNPs + 50 mg BNPs/kg soil (50V + 50B). Based on ERT results, for biochemical endpoints assessment non-lethal experimental conditions were selected (10B, 10V and 10B + 10V), including also the C and SC.

Soil spiking was performed according to Barreto and co-workers [18] (details can be found in the Suplementary Material).

#### 2.3.1. Enchytraeid Reproduction Test (ERT)

ERT was performed following OECD guidelines with minor modifications [31]. Briefly, synchronized age cultures of *E. crypticus* were previously prepared according to Barreto and co-workers [18]. Per replicate, 10 juveniles (17–19 days old) were introduced in 4 of the 5 replicates of each experimental group (C, SC, 10B, 50B, 10V, 50V, 10V + 10B, 10V + 50B, 50V + 10B and 50V + 50B), 1 replicate was left without organisms to measure pH at the beginning and end of the test. The organisms were fed once per week. After 28 days, survival (number of adults) and reproduction (number of juveniles) were evaluated, as described in Barreto and co-workers [18].

#### 2.3.2. Biochemical Assays

Biochemical endpoints were evaluated at different exposure times (3, 7 and 14 days), applying spectrometric methods (Thermo Scientific, Multiskan Spectrum microplate reader). Per treatment, 16 replicates were performed (5 per sampling time and 1 without organisms for pH measurement). *E. crypticus* adults with well-developed clitellum were selected and introduced in each vessel (50 organisms/replicate). The organisms were fed on the first day of the experiment and at day 7 with 33 mg of autoclaved ground oats and maintained at 20 ± 1 °C with a 16 h:8 h (light:dark) photoperiod. At each sampling time, the organisms of 5 replicates (per condition) were removed from the soil, frozen in liquid nitrogen, and stored at −80 °C until further analysis.

Potassium phosphate buffer (1 mL, 0.1 mM, pH 7.4) was added to each replicate prior to homogenization with an ultrasonic homogenizer (Sonifier 250, Branson sonicator). To 150 μL of the homogenate was added 2.5 μL BHT (2,6-dieter-butyl-4-metylphenol) 4% in methanol and stored at −80 °C for further lipid peroxidation (LPO) determination. The remaining homogenate was centrifuged at 10,000× *g* for 20 min at 4 °C. The resultant supernatant (PMS-post-mitochondrial supernatant) was collected and divided for each biochemical endpoint (catalase (CAT), glutathione reductase (GR), glutathione S-transferase (GST) and acetylcholinesterase (AChE)) and stored at −80 °C until further analysis. Protein content of the homogenate and PMS was assessed using the Bradford method [32], using bovine *γ*—globuline as a standard.

LPO levels were determined in the organisms homogenate according to Ohkawa and co-workers (1979), Bird and Draper (1984), Wilhelm and co-workers (2001) [33,34,35]: absorbance was measured at 535 nm and LPO expressed as μmol of thiobarbituric acid reactive substances (TBARS) formed per milligram of protein (*ε* = 1.56 × 10^5^ M^−1^·cm^−1^). CAT activity was measured according to Claiborne (1985) and Giri and co-workers (1996) [36,37]: absorbance was measured at 240 nm and translated to CAT activity in terms of µmol hydrogen peroxide (H_2_O_2_) consumed per min per mg of protein (*ε* = 40 M^−1^·cm^−1^). GR activity (mmol/min/mg protein) was determined according to Cribb and co-workers (1989) [38]: the decrease in Nicotinamide Adenine Dinucleotide Phosphate (NADPH) was measured at 340 nm (*ε* = 6.22 × 10^3^ M^−1^·cm^−1^). GST activity was determined according to Habig and co-workers (1974) [39]: absorbance at 340 nm was recorded and translated to GST activity presented as nmol GS-DNB conjugate per min per mg of protein (*ε* = 9.6 × 10^3^ mM^−1^·cm^−1^). AChE activity was determined by the Ellman (1961) method with adaptation of according to Guilhermino and co-workers (1996) [40,41]: absorbance at 414 nm was recorded and translated to AChE activity in terms of mmol/min/mg protein (*ε* = 13.6 × 10^3^ M^−1^·cm^−1^).

### 2.4. Data Analysis

Prior to further data analysis, differences between C and SC were evaluated using a Student *t*-test. C and SC were pooled and analyzed together as control (0) according to OECD guidelines [31,42].

Differences in survival and reproduction between groups were analyzed using non-parametric Kruskal-Wallis analysis of variance (ANOVA) followed by a Dunn’s test versus control group (parametric criteria not achieved). Differences in biochemical endpoints by time and group were analyzed using one-way ANOVA followed by a Tukey post hoc test when parametric criteria (normality and homogeneity of variance) were achieved, and, if not, a non-parametric Kruskal-Wallis ANOVA was conducted followed by a Dunn’s test. For all the data analysis, *p* < 0.05 was considered statistically significant. All statistical analysis and graphics were performed using Sigma Plot 12.5 software (Munich, Germany). Outliers were verified by the outlier labelling rule [43]. Principal component analysis (PCA) was executed in the R-studio software using the function prcomp from the stats package and the ggbiplot function from the ggplot2 package for the several biochemical endpoints in each time point (3, 7 and 14 days).

## 3. Results

### 3.1. Characterization of the Test Materials

The BNPs (10B and 50B) and VNPs (10V and 50V) test dispersions presented an average HS of 96 (PDI: 0.25) and 87 (PDI: 0.097) nm and an average ZP of −37.8 and −20.6 mV, respectively (Figure S1). While the BNPs + VNPs dispersions presented two distinct peaks, the peak with the higher intensity showed an average HS of 938 nm and the one with the lower intensity of 187 nm. The average ZP of BNPs + VNPs dispersions ranged from −38.4 to −22.5 mV, depending on the relative concentrations of VNPs and BNPs (Figure S1).

### 3.2. Enchytraeid Reproduction Test (ERT)

During the ERT the soil pH was maintained between reference values [31]. No significant differences were detected between C and SC (*p* > 0.05), a pooled control (C + SC) was used in subsequent analysis [31,42].

**Figure 1:**
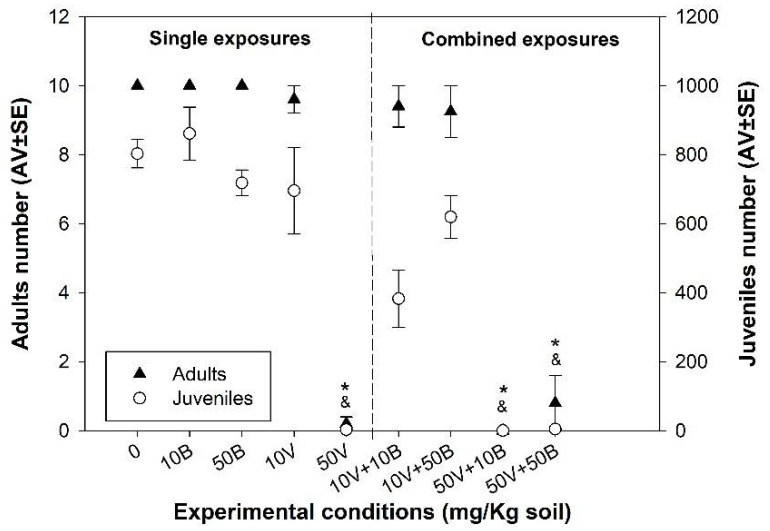
Effects on *Enchytraeus crypticus* survival (adult number) and reproduction (juvenile number) after 28 days exposure to: 0—control and solvent control; 10B—10 mg BNPs/kg soil; 50B—50 mg BNPs/kg soil; 10V—10 mg VNPs/kg soil; 50V—50 mg VNPs/kg soil; 10V + 10B—10 mg VNPs + 10 mg BNPs/kg soil; 10V + 50B—10 mg VNPs + 50 mg BNPs/kg soil; 50V + 10B—50 mg VNPs + 10 mg BNPs/kg soil; 50V + 50B—50 mg VNPs + 50 mg BNPs/kg soil, in LUFA 2.2 soil. Results are expressed as average value (AV) ± standard error (SE) (*n* = 4). *—Significant differences in the number of adults compared to control (*p* < 0.05); &—Significant differences in the number of juveniles compared to control (*p* < 0.05).

While no significant alterations (*p* > 0.05) in *E. crypticus* survival and reproduction were observed after exposure to BNPs (10B or 50B), a decrease (*p* < 0.05) in organism survival and reproduction was observed for single VNPs (50V) and in the combination (50V + 10B or 50B) (Figure 1).

### 3.3. Biochemical Assays

Assessed biochemical endpoints were those involved in processes such as oxidative stress/damage, detoxification (LPO, CAT, GR and GST) and neurotransmission (ChE). The different endpoints were evaluated at three distinct times 3, 7 and 14 days. No significant differences were detected between C and SC (*p* > 0.05), and a pooled control (C + SC) was used in subsequent analysis [31,42].

#### 3.3.1. Oxidative Stress/Damage and Detoxification

**Figure 2:**
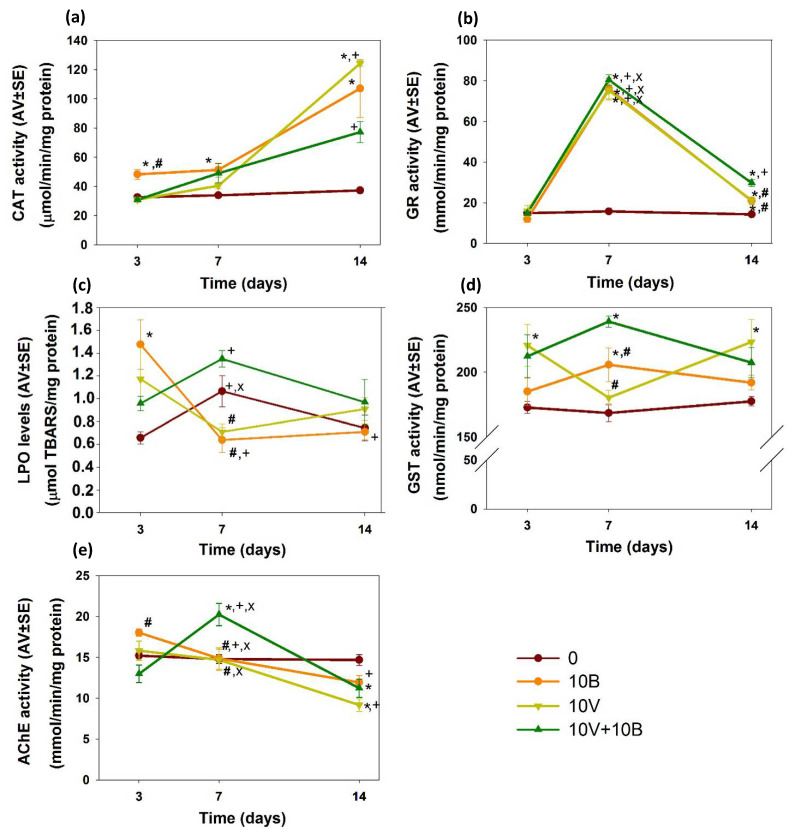
Effects on (**a**) catalase (CAT), (**b**) glutathione reductase (GR), (**c**) lipid peroxidation (LPO) levels, (**d**) glutathione-s-transferase (GST) and (**e**) acetylcholinesterase (AChE) activities of *Enchytraeus crypticus* exposed during 3, 7 and 14 days to: 0—control and solvent control; 10B—10 mg BNPs/kg soil; 10V—10 mg VNPs/kg soil; 10V + 10B—10 mg VNPs + 10 mg BNPs/kg soil, in LUFA 2.2 soil. Results are expressed as average values (AV) ± standard errors (SE) (*n* = 5). Significant differences (*p* < 0.05) relative to: (*) control group; (#) 10V + 10B; (+) day 3; (x) day 14.

An increase (*p* < 0.05) in CAT activity, in relation to the control group, was observed in the organisms exposed to 10B at days 3, 7 and 14. After 14 days, the organisms exposed to 10V also presented an increase (*p* < 0.05) in CAT activity, compared with the control group (Figure 2a).

At days 7 and 14 an increase (*p* < 0.05) in GR activity was observed in the organisms exposed to 10V, 10B and 10V + 10B, compared with the control group. Moreover, at day 14 the organisms exposed to 10V + 10B presented an increase (*p* < 0.05) in GR activity not only in relation to the control group but also in relation to the single exposures (Figure 2b).

At day 3, LPO levels increased (*p* < 0.05) in the organisms exposed to BNPs in relation to the control group, while at day 7 and 14 no significant alterations (*p* > 0.05) were detected. However, at day 7 the organisms exposed to single BNPs and VNPs presented lower LPO levels compared with the organisms exposed to the combination of both (Figure 2c).

An increase (*p* < 0.05) in GST activity was observed at days 3 and 14 in the organisms exposed to 10V, relative to the control group. Moreover, at day 7, the organisms exposed to 10B and 10V + 10B presented an increase (*p* < 0.05) in GST activity compared to the control group, while the organisms exposed to 10V presented lower activity (*p* < 0.05) compared to 10V + 10B (Figure 2d).

#### 3.3.2. Neurotoxicity

At day 3, the organisms exposed to 10B revealed higher AChE activity (*p* < 0.05) in relation to combination NPs. At day 7, this relation was inverted with an increase of AChE activity (*p* < 0.05) in the organisms exposed to 10V + 10B compared to single exposures and the control group. At day 14, in relation to the control group, a decrease of AChE activity (*p* < 0.05) was detected in the organisms exposed to 10V and 10V + 10B (Figure 2e).

#### 3.3.3. Principal Component Analysis (PCA)

**Figure 3:**
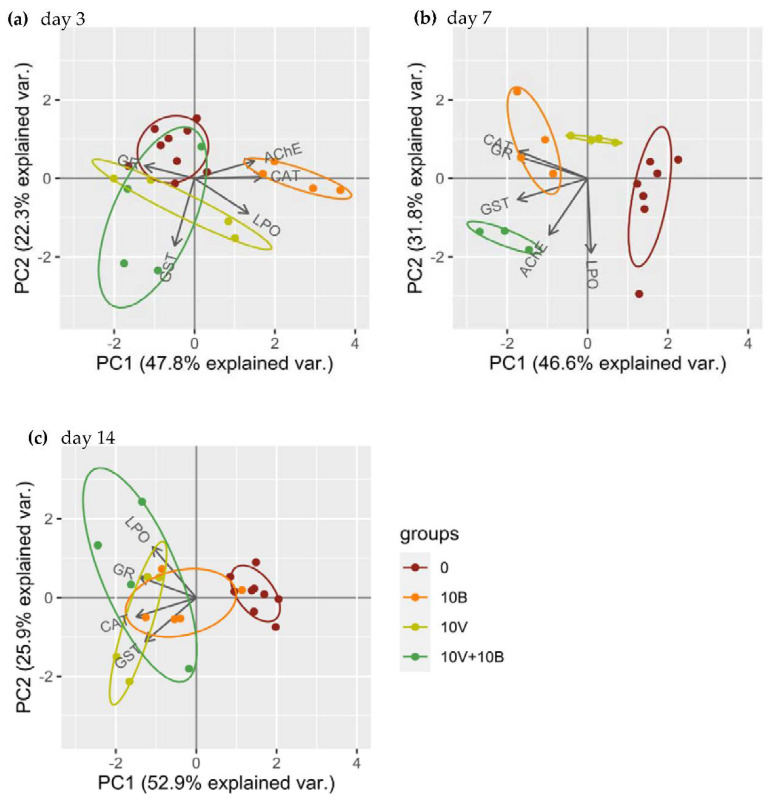
Principal component analysis (PCA) of the biochemical endpoints: catalase (CAT), glutathione reductase (GR), glutathione-s-transferase (GST) and acetylcholinesterase (AChE) activities; and lipid peroxidation (LPO) levels related with the different tested exposures at days: (**a**) 3; (**b**) 7; and (**c**) 14. 0—control and solvent control; 10B—10 mg BNPs/kg soil; 10V—10 mg VNPs/kg soil; 10V + 10B—10 mg VNPs + 10 mg BNPs /kg soil.

Together, the first two components (PC1 and PC2) account for 70.1, 78.4 and 76.7% of biochemical data variability at days 3, 7 and 14, respectively (Table S2). The score plots present distinct response patterns for the control (0), VNPs (10V) and BNPs (10B) at all times. The responses in the combination VNPs + BNPs (10V + 10B) group are dispersed at day 3 and 14 (Figure 3). At day 3, CAT is positively correlated with AChE (<90° angle) and negatively with GR (angle of approximately 180°), while GST has no/low correlation with the other variables. BNPs are mainly associated with AChE and CAT variables (Figure 3a). At day 7, CAT is highly correlated with GR while the other variable presents a low level of correlation. BNPs are mainly associated with CAT and GR variables while 10V + 10B is mainly associated with AChE and GST (Figure 3b). At day 14, CAT is positively correlated with GST and negatively with AChE, while LPO is positively correlated with GR. The control is mainly associated with AChE variable (Figure 3c).

## 4. Discussion

The growing use of NMs has encouraged the development of a new area in the toxicology field, nanotoxicology. However, the evaluation of effects of nano-contaminants has presented different and new challenges when compared with traditional contaminants, such as metals, pharmaceuticals, solvents and surfactants [44]. Several NP characteristics, such as size, aggregation, shape and surface charge, influence their bioavailability and toxicity [2,4,9,12]. The performed characterization showed that NPs’ test dispersions (VNPs and BNPs) presented average sizes indicated by the supplier (80–100 nm). However, in combination (VNPs + BNPs) they were found to give rise to an increase in the HS, suggesting processes of NPs’ aggregation and/or agglomeration. The aggregation between particles is known as hetero- or homo-aggregation, based on the differences or similarity between the particles. This aggregation increases NPs’ size, potentially altering the uptake and toxicity of the NPs [45]. The ZP value analyses indicated a negative surface charge of the single VNP (−20.6 mV) and BNP (−37.8 mV) test dispersions. For VNPs + BNPs test dispersions, ZP values were dependent on which type of NP was predominant. In general, ZP values lower than −30 mV indicate a stable nanosuspension; if all the particles in a suspension have a strong negative ZP, they repel one another and have no tendency to aggregate or agglomerate [46]. However, based on the HS obtained for VNPs + BNPs test dispersions, it seems that when combined, the tested NPs become clustered into larger structures.

Barreto and co-workers [18] evaluated *E. crypticus* survival and reproduction after 28 and 56 days of exposure to VNPs or BNPs (concentrations ranging between 10 to 500 mg/kg). After 28 days, they observed a decrease in the survival of the organisms exposed to 50, 100 and 500 mg VNPs/kg and in reproduction in the organisms exposed to 500 mg BNPs/kg and 10, 50, 100 and 500 mg VNPs/kg. Interestingly, the extension to 56 days revealed a higher toxicity of BNPs with a decrease in the juvenile number at 100 and 500 mg/kg and a lower toxicity of VNPs with the decrease in the juvenile number only detected in concentrations higher than 50 mg/kg [18]. The higher toxicity of VNPs compared with BNPs is in line with observed toxicity of their bulk counterparts [23,24,26,47,48]. Previous works have already reported on the production of ROS in mammals (in vivo and in vitro) due to VNPs [20,21] and detected indications of increased ROS production (decrease of total glutathione (TG) content and GST activity) in *D. rerio* exposed to VNPs and BNPs [19].

Ecosystems are exposed to a mixture of contaminants that can exert synergistic, antagonistic, additive or independent effects [49]. Existing information regarding the effect of NP mixtures is very scarce. However, different interaction effects have already been reported. The exposure of *D. magna* to NPs of CuO (copper oxide) and ZnO (zinc oxide) induced a higher toxicity than expected based on individual counterparts (synergistic effect) [49,50], while, for example TiO_2_ (titanium dioxide) NPs + CeO_2_ (cerium dioxide) NPs or TiO_2_ NPs + Fe_3_O_4_ (iron(II,III) oxide) NPs exerted antagonistic effects [49,51,52]. Our work demonstrated, as previously reported, that VNPs (single exposure) impaired reproduction and survival at lower concentrations than BNPs (single exposure) [18]. However, while Barreto and co-workers observed a decrease in organisms’ reproduction after 28 days in the presence of 10 mg VNPs/kg, in our work, a significant decrease was only observed at 50 mg VNPs/kg, similar to the reported effect after 56 days of VNP exposure [18]. The exposure to VNPs + BNPs resulted in similar effects as the individual ones. A decrease in organism survival and reproduction was observed in the combinations that included 50 mg VNPs/kg, indicating that the presence of BNPs (10 or 50 mg/kg) did not interfere with the effects caused by VNPs. Moreover, the formation of NP aggregates/agglomerates in VNPs + BNPs suspensions did not modify the toxicity of the VNPs to *E. crypticus*. The results indicate that, concerning organism survival and reproduction, when in a mixture, VNPs and BNPs present an effect of addition or independent action [49].

The interaction of NPs with the organisms’ defense systems seems almost transversal. We can easily find NPs reported as scavengers of ROS (antioxidant NPs) or NPs able to generate ROS [4,6,53,54,55]. The equilibrium between oxidative and reducing reactions is fundamental to the proper activity of the cells, since increase in ROS levels generates misfolded proteins, oxidized lipids and DNA damage [56,57]. The accumulation of oxidized lipids, considered one of the major mechanisms involved in oxidative cell damage, disrupt membrane stability, inhibit protein synthesis and enzyme activity, disrupt biological homeostasis and potentially result in cell death [56,57,58,59]. To respond to the oxidative stress caused by the increase in ROS, cells present several antioxidant defense mechanisms, mainly constituted by free radical scavengers and specific proteins (such as CAT, GR and TG) [56,58,60]. The NPs evaluated in this study (VNPs and BNPs) were previously reported to decrease TG content and GST activity in *D. rerio* suggesting an increase in ROS production [19]. However, the bulk counterparts together exerted an antagonistic response with the B protecting the cells from the oxidative stress produced by V exposure [25]. To shed light on the oxidative/antioxidant effects of BNPs and VNPs, we chose the no-effect concentration of 10 mg/kg and evaluated the effects in several oxidative stress markers after 3, 7 and 14 days of exposure. Increase in the LPO levels is a harmful consequence of increase in ROS levels [61,62] and this was seen early for BNP exposure (3 days). Indeed, the antioxidant and detoxification systems (CAT, GST, GR and GST) were ineffective, even with CAT activity increase at day 3. However, the posterior reinforcement (7 days) of the antioxidant defense (CAT activity increase associated with GST and GR activities increase) allowed compensation for the initial ROS imbalance, indicating an effective response to counteract the increase of ROS levels. Further increase in GST and GR activities may indicate that CAT, together with other enzymes, was able to remove hydroperoxides and maintain the redox balance (seen as LPO levels decrease). Overall, BNP exposure induced an early increase in *E. crypticus* lipid damage but the activation of the antioxidant and detoxification mechanisms restored the redox equilibrium and decreased the LPO levels to values similar to the ones found in the control organisms.

The LPO levels in the organisms exposed to VNPs were similar to the levels detected in the control group, suggesting that detoxification and antioxidant mechanisms were able to cope with contamination. Unlike the organisms exposed to BNPs, the first mechanism detected to be upregulated (3 days) in the ones exposed to VNPs was the antioxidant and detoxification enzyme, GST. GR activity was further activated (7 days) since educed glutathione (GSH, used by GST) needed to be renewed in the cell by the GSH redox cycling. The GSH may also be used as a scavenger thiol of ROS, indicating hydroperoxide removal [63]. However, CAT activity was also required to block ROS because later activation was also found. In general, *E. crypticus* exposed to VNPs presented latter activation of the first barrier of the antioxidant mechanism (CAT activity increased at day 14) than ones exposed to BNPs (CAT activity increased at day 3). Lung cells exposed to VO_2_ (vanadium dioxide) NPs showed ROS production and decreased GSH [20]. The dissimilar “choice” of antioxidant system to react against ROS (day 3: elevated GST and CAT for VNPs and BNPs, respectively) can be explained by the chemical nature of the two NPs. Generally, metallic NPs, such as VNPs, are more stable and less prone to dissolution, compared with oxide versions (VO_2_ NPs) [64]. Consequently, metallic oxide NPs are more likely to cause ROS formation and oxidative stress [64]. Although VNPs induced the activation of detoxification and antioxidant mechanisms (GST, CAT and GR activities), the lack of LPO suggests the existence of other toxicity mechanisms to explain the high level of toxicity detected in the survival and reproduction of organisms exposed to VNPs. Future studies should explore the possibility of a direct interaction between NPs and DNA molecules, with possible inhibition of replication and transcription, or even possible causing of chromosomal breakage [9].

The biochemical responses presented an interesting picture for organisms exposed to BNPs + VNPs. Although, regarding survival and reproduction, the BNPs and VNPs appear to have an independent or additive effect, the same was not observed in relation to oxidative stress/damage. In the presence of VNPs, BNPs did not cause an increase in LPO levels, even at day 3, when no activation of the antioxidant mechanism was detected, suggesting an antagonistic effect. However, compared with the control, the increase in GR activity, at day 7, indicates compensation due to ROS formation, similar to the organisms exposed to VNPs or BNPs. Moreover, at day 14, GR activity increased in the organisms exposed to BNPs + VNPs (versus control and single NPs), possibly to counteract increase in hydroperoxide amounts. Despite the raised tendency of CAT activity, the principal enzyme in hydroperoxide reduction [65], this was not preponderant in the decrease of the excessive hydroperoxide formed. The GST activity at day 7, similar to the observation in the organisms exposed to BNPs, indicates the activation of detoxification mechanisms.

*E. crypticus* biochemical responses to the toxicity exerted by BNPs and VNPs are dependent on the time and type of exposure (single versus combination), with specific antioxidant mechanisms activated in different moments in the presence of different NPs, as highlighted by the PCA analysis. The individualized clusters in the PCA analysis for the control, VNP and BNP groups emphasize the different level and coordination of the biochemical response observed. These results are in line with the alterations observed through time in the levels, or activity levels, of CAT, GR, LPO and AChE, mainly in organisms exposed to NPS (single and combined). A time-dependent response of the antioxidant mechanisms was previously reported in enchytraeids exposed to Cu (copper) and Ag (silver) NPs [66,67]. Overall, the antioxidant response observed in *E. crypticus* exposed to BNPs + VNPs is not as robust as the response in organisms exposed to BNPs or VNPs, but is enough to avoid the increase in LPO levels. Therefore, the higher toxicity of VNPs single or in the presence of BNPs, on the survival and reproduction of *E. crypticus*, can be related to a delay in the activation of first line antioxidant defenses, as previously reported by Ribeiro and co-workers [66], or with different toxicity mechanisms, such as a direct interaction with DNA molecules [9].

Besides the decrease in survival and reproduction, Barreto and co-workers also reported avoidance behavior in *E. crypticus* exposed to 10 mg VNPs/kg. However no avoidance behavior was detected in the organisms exposed to BNPs [18]. Inhibition of the neurotransmitter AChE has been linked to the absence of avoidance behavior [18,68]. The neurotransmitter AChE cleaves the acetylcholine released in the synaptic cleft ending signal transmission, inhibition of AChE causes over-stimulation while the activation is observed in apoptotic cells or blockage of the cholinergic receptors [61,69]. To evaluate potential neurotoxicity caused by BNPs and/or VNPs we considered alterations in AChE activity. Our results were in line with the observations in *D. rerio* [19]; the activity of AChE decreased after 14 days in the *E. crypticus* exposed to VNPs and no relevant alteration was observed in the organisms exposed to BNPs. Thus, the absence of the avoidance behavior observed by Barreto and co-workers [18] may be related to the up-regulation of the gamma-aminobutyric acid system or even to the low concentrations tested [18,70]. Interestingly, in the organisms exposed to VNPs + BNPs the response observed in AChE varied with time and showed different timing in response compared with VNPs. To sum up, in the tested concentrations, VNPs caused neurotoxicity (AChE activity decrease); as a mixture, BNPs were not able to prevent the toxic effects of VNPs.

## 5. Conclusions

The presented study evaluated the impact of VNPs, BNPs and the combination of both on the reproduction, survival and biochemical responses of the soil organism *E. crypticus*. VNPs presented a higher toxicity than BNPs, decreasing *E. crypticus* survival and reproduction. At an individual level, the effects of VNPs + BNPs seem to be additive or independent. However, the biochemical responses were specific to time, NPs and exposure (single and combination). Faster and stronger response was detected in organisms exposed to BNPs > VNPs > VNPs + BNPs. Neurotoxicity was only detected in the treatments containing VNPs. The current study shows that BNPs had no effect on the toxicity of VNPs, despite the trigger of antioxidant and detoxification mechanisms through time and increased LPO levels on day 3. Further studies should explore the hypothesis of VNPs to exert toxicity directly through DNA; for example, regulating gene expression or causing DNA damage.

## Data Availability

The data are available on request from the corresponding author.

## Supplementary Materials

The following supporting information can be downloaded at https://www.mdpi.com/article/10.3390/nano12091478/s1. Figure S1: Zeta potential, hydrodynamic size and polydispersity index of nanoparticles test dispersions (prepared in ultrapure water).; Tables S1 and S2: LUFA 2.2 standard natural soil main characteristics and PCA components. Reference [71] is cited in the supplementary materials.
