# Supplementary material for: Single and Mixture Toxicity of Boron and Vanadium Nanoparticles in the Soil Annelid Enchytraeus crypticus: A Multi-Biomarker Approach
Source: Nanomaterials (Basel). 2022 Apr 27;12(9):1478. doi: 10.3390/nano12091478 (PMC9100768; doi:10.3390/nano12091478)
Supplement: Supplementary file 1 [file nanomaterials-12-01478-s001.zip › nanomaterials-1680298-supplementary.pdf]

Supplementary Material

Single and Mixture Toxicity of Boron and Vanadium Nanoparticles in the Soil Annelid *Enchytraeus crypticus*: A Multi-Biomarker Approach

Ana Capitão †, Joana Santos †, Angela Barreto, Mónica J. B. Amorim and Vera L. Maria *

Department of Biology & CESAM, University of Aveiro, 3810-193 Aveiro, Portugal; amcapitao@ua.pt (A.C.); joanasilvasantos@ua.pt (J. S.); abarreto@ua.pt (A. B.); mjamorim@ua.pt (M. J. B. A.)
* Correspondence: vmaria@ua.pt; Tel.: +351-234-370-350; Fax: +351-234-372-587
† These authors contributed equally to this work.

Citation: Capitão, A.; Santos, J.; Barreto, A..; Amorim, M. J. B.; Maria, V. L. Single and Mixture Toxicity of Boron and Vanadium Nanoparticles in the Soil Annelid *Enchytraeus crypticus*: A Multi-Biomarker Approach. *Nanomaterials* **2022**, *12*, 1478. https://doi.org/10.3390/nano12091478

Academic Editors: Julian Blasco and Ilaria Corsi

Received: 29 March 2022
Accepted: 23 April 2022
Published: 27 April 2022

Publisher's Note: MDPI stays neutral with regard to jurisdictional claims in published maps and institutional affiliations.

Copyright: © 2022 by the authors. Licensee MDPI, Basel, Switzerland. This article is an open access article distributed under the terms and conditions of the Creative Commons Attribution (CC BY) license (https://creativecommons.org/licenses/by/4.0/).

Spiking soil procedure

The soil was dried for 48h at 60ºC prior to use. The control was prepared adding deionized water, to reach 50% of the WHC (water holding capacity), to the dry soil. The presence of Triton X-100 (2%) in the stock solutions required the preparation of a solvent control. A stock solution of 2% Triton X-100 was prepared for the solvent control. Aqueous working solutions of the solvent and NPs were added to the pre-moistened soil of each replicate, ensuring a final content of 50% of the soil WHC, in the end individual replicates were mixed thoroughly [31,71]. The test started 1 day after the spiking procedures.

Table S1. LUFA 2.2 standard natural soil main characteristics.

**Batch nº**	Sp 222319
**Sampling date**	03/06/19
**Soil type (USDA)**	Sandy loam
**Maximum water holding capacity (g/100g)**	44.8±2.9
**Organic carbon in % C**	1.71±0.30
**Nitrogen in %N**	0.18±0.03
**pH-value (0.01M $CaCl_2$)**	5.6±0.4
**Cation exchange capacity (meq/100g)**	9.2±1.4

Table S2. PCA components.

3 days					
	**PCA1**	**PCA2**	**PCA3**	**PCA4**	**PCA5**
**Standard deviation**	1.5455	1.0563	0.8329	0.7242	0.5268
**Proportion of Variance**	0.4777	0.2231	0.1387	0.1049	0.0555
**Cumulative Proportion**	0.4777	0.7009	0.8396	0.9445	1.0000
**7 days**					
	**PCA1**	**PCA2**	**PCA3**	**PCA4**	**PCA5**
**Standard deviation**	1.5265	1.2617	0.7161	0.5917	0.4636
**Proportion of Variance**	0.4661	0.3184	0.1026	0.0700	0.0429
**Cumulative Proportion**	0.4661	0.7844	0.8870	0.9570	1.0000
**14 days**					
	**PCA1**	**PCA2**	**PCA3**	**PCA4**	**PCA5**
**Standard deviation**	1.6671	1.0280	0.7621	0.5789	0.4981
**Proportion of Variance**	0.5559	0.2113	0.1162	0.0670	0.0496
**Cumulative Proportion**	0.5559	0.7672	0.8834	0.9504	1.0000

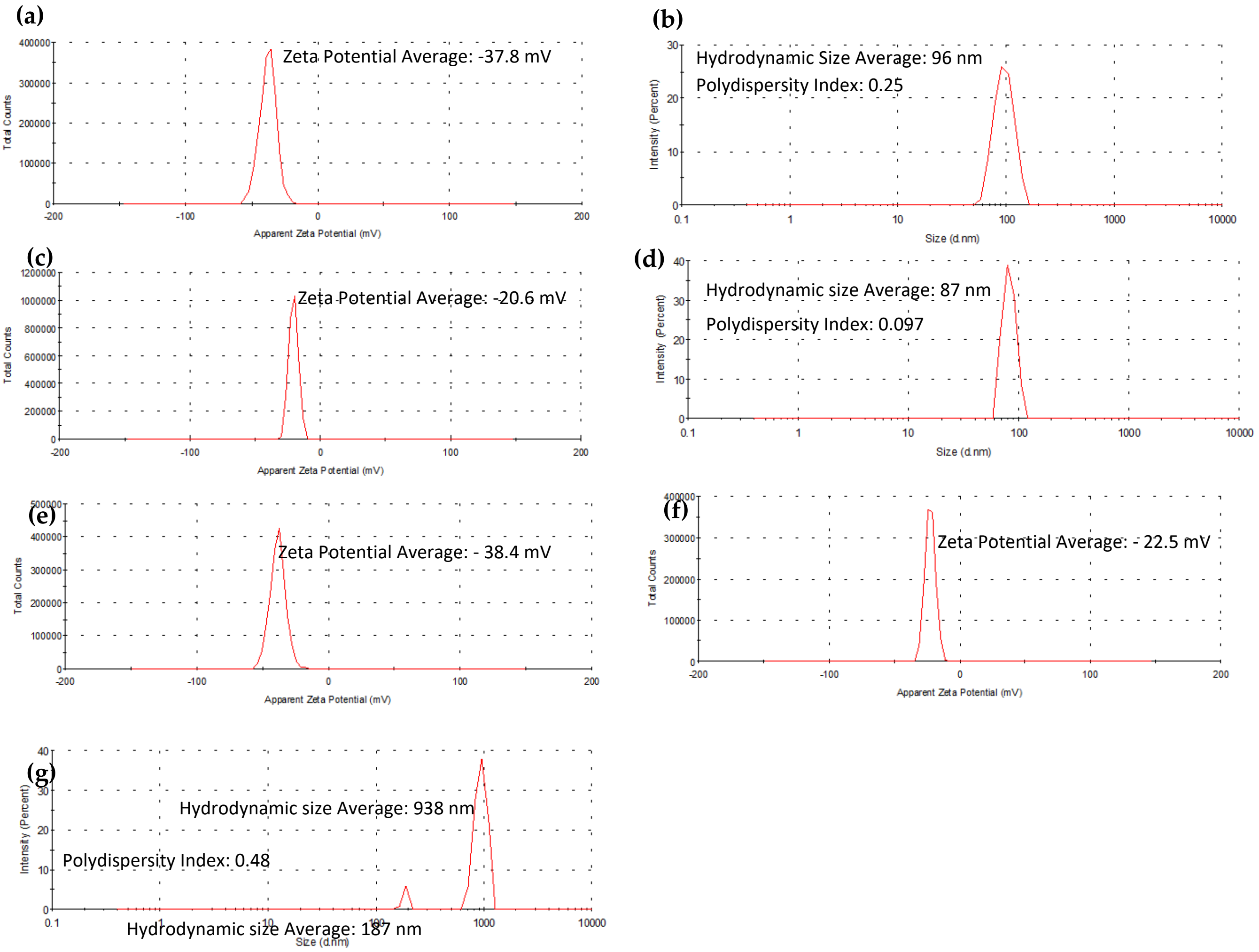

Figure S1. Zeta potential, hydrodynamic size and polydispersity index of nanoparticles test dispersions (prepared in ultrapure water). **(a)** Zeta potential of boron nanoparticles dispersions (10 or 50mg/Kg); **(b)** Hydrodinamic size of boron nanoparticles dispersions (10 or 50mg/kg); **(c)** Zeta potential of vanadium nanoparticles dispersions (10 or 50mg/kg); **(d)** Hydrodinamic size of vanadium nanoparticles disperions (10 or 50mg/kg); **(e)** Zeta potential of boron nanoparticles + vanadium nanoparticles dispersions (when the concentration of boron nanoparticles (50mg/kg) > concentration of vanadium nanoparticles (10mg/kg) and when the concentration is the same for both types of nanoparticles (10 or 50mg/kg); **(f)** Zeta potential of boron nanoparticles + vanadium nanoparticles (when the concentration of vanadium nanoparticles (50mg/kg) > concentration of boron nanoparticles (10mg/kg)); **(g)** Hydrodinamic size of boron nanoparticles (10 or 50mg/kg) + vanadium nanoparticles (10 or 50mg/kg).

References

31. Organization for Economic Co-operation and Development (OECD). *Test No. 220: Enchytraeid Reproduction Test*; OECD Publishing: Paris, France, 2004.
71. OECD. *OECD Series on the Safety of Manufactured Nanomaterials, No. 36: Guidance on Sample Preparation and Dosimetry for the Safety Testing of Manufactured Nanomaterials*; OECD: Paris, France, 2012.
